# A Case of Extra-nodal Rosai-Dorfman Disease

**DOI:** 10.7759/cureus.90643

**Published:** 2025-08-21

**Authors:** Abir Islam, James P Smith, Gowrish Vaka, Wasifuddin Syed, Roopin P Singh, Hassan Khanani

**Affiliations:** 1 Internal Medicine, Edward Via College of Osteopathic Medicine, Monroe, USA; 2 General Surgery, St. Francis Hospital, Monroe, USA; 3 Medical School, Edward Via College of Osteopathic Medicine, Monroe, USA; 4 Medicine, Willis Knighton Health, Shreveport, USA

**Keywords:** computed tomography, deep soft tissue mass, destombes–rosai–dorfman disease, extranodal sinus histiocytosis with massive lymphadenopathy, magnetic resonance imaging, non-langerhans cell histiocytosis, rosai-dorfman disease, rosai dorfman syndrome, sinus histiocytosis with massive lymphadenopathy, spine

## Abstract

Rosai-Dorfman disease (RDD) doesn’t cause major health issues for most people. That said, it can present in many different ways and often resembles more serious conditions, including certain types of cancer. Because RDD is so rare, it is often overlooked, partly due to the absence of clinical algorithms for classifying its subtypes and guiding management. Thus, it is significant to consider advancements in the various clinical manifestations of RDD, which can effectively guide concise clinical management.

This case report will discuss a 50-year-old female who had a soft tissue mass for over 15 years, which recently began to enlarge exponentially over the course of a few months. CT imaging revealed a cutaneous mass on the left flank. The initial diagnosis was leaning towards a lipoma. However, a core needle biopsy was performed and examined histopathologically to rule out malignancy. The findings of the biopsy confirmed the diagnosis of extra-nodal cutaneous RDD by using specific immunohistochemistry markers. The patient underwent a wide, local surgical excision, with resolution of symptoms.

This case shows an unusual form of RDD that appears outside the lymph nodes, specifically in the skin's soft tissues. Though skin manifestations can be seen in extra-nodal RDD, the majority of RDD is confined to the lymph nodes. This case presents an opportunity to expand knowledge of a rare benign disease that presents with diverse clinical manifestations that can mimic a lipoma or malignancy (lymphoma), enabling clinicians to accurately diagnose and differentiate it from malignancies that may have similar presentations.

## Introduction

Rosai-Dorfman disease (RDD), also known as sinus histiocytosis with massive lymphadenopathy, is a rare form of non-Langerhans cell histiocytosis (non-LCH). Non-LCH is a type of sinus histiocytosis that can affect different parts of the body, but does not meet the criteria for the diagnosis of Langerhans cell histiocytosis (LCH) [1]; it falls under the “R” group of non-LCH. The "R" group stands for Rosai-Dorfman since it falls under the umbrella of non-LCH. This disease garners significant interest in the medical field due to its diverse clinical manifestations and its rarity.

RDD classically occurs when there is an accumulation of histiocytes in the lymph nodes [2]. Histiocytes are a subset of immune cells comprised of macrophages, neutrophils, and lymphocytes. These immune cells can often be found in circulation and pass through lymph nodes as they respond to antigens. The majority of nodal (classic) cases include lymphadenopathy in the cervical lymph nodes; however, extra-cervical lymph nodes and extra-nodal parts of the body may also be affected [3]. RDD affects extra-nodal sites in about 40% of patients, consisting of, but not limited to, soft tissue, sinuses, salivary glands, bones, eyes, nasal cavities, and skin (cutaneous) [4].

RDD is a rare benign disease. It is most commonly diagnosed in the first 10 years of life. About one in every 200,000 people are diagnosed with RDD [4]. The etiology of RDD is currently unknown and considered idiopathic. No certain etiology has been established thus far, but there have been connections between a few conditions and RDD. This disease has been found to be associated with viral infections (herpes simplex virus, Epstein-Barr virus, cytomegalovirus), cancer (Hodgkin's/non-Hodgkin’s lymphoma), and autoimmune conditions (systemic lupus erythematosus, juvenile idiopathic arthritis, autoimmune hemolytic anemia) [5].

Symptoms of RDD depend primarily on the site of the body affected. Classic nodal RDD, which affects the cervical nodes, will present with painless and swollen neck lumps [6]; lymphadenopathy is one of the most common clinical presentations. Other presentations include non-specific general findings, such as night sweats, fever, shortness of breath, weight loss, etc. Cutaneous Rosai-Dorfman Disease, or extra-nodal RDD, may appear anywhere on the body. Physical manifestations can have a wide variety of findings. Findings can possibly range from asymptomatic, painless tissue swelling to flat or raised, pus-filled or solid, discolored skin lesions [7]. Lab markers will typically show elevated erythrocyte sedimentation rates, accompanied by possible leukocytosis and fever [8].

Visualization of skin lesions and swollen lymph nodes on physical exam presents a wide differential. Imaging, such as X-rays, ultrasound, MRI, and CT/PET scans, is particularly useful to localize the extent of body involvement. Definite diagnosis is usually made with biopsy of a swollen lymph node or tissue sample, which is sent to the pathologist for confirmation of disease with immunohistochemistry/molecular testing and interpretation.

This article was previously presented as a poster presentation at the American College of Osteopathic Surgeons (ACOS) Winter Symposium in 2024.

## Case presentation

**Figure 1:**
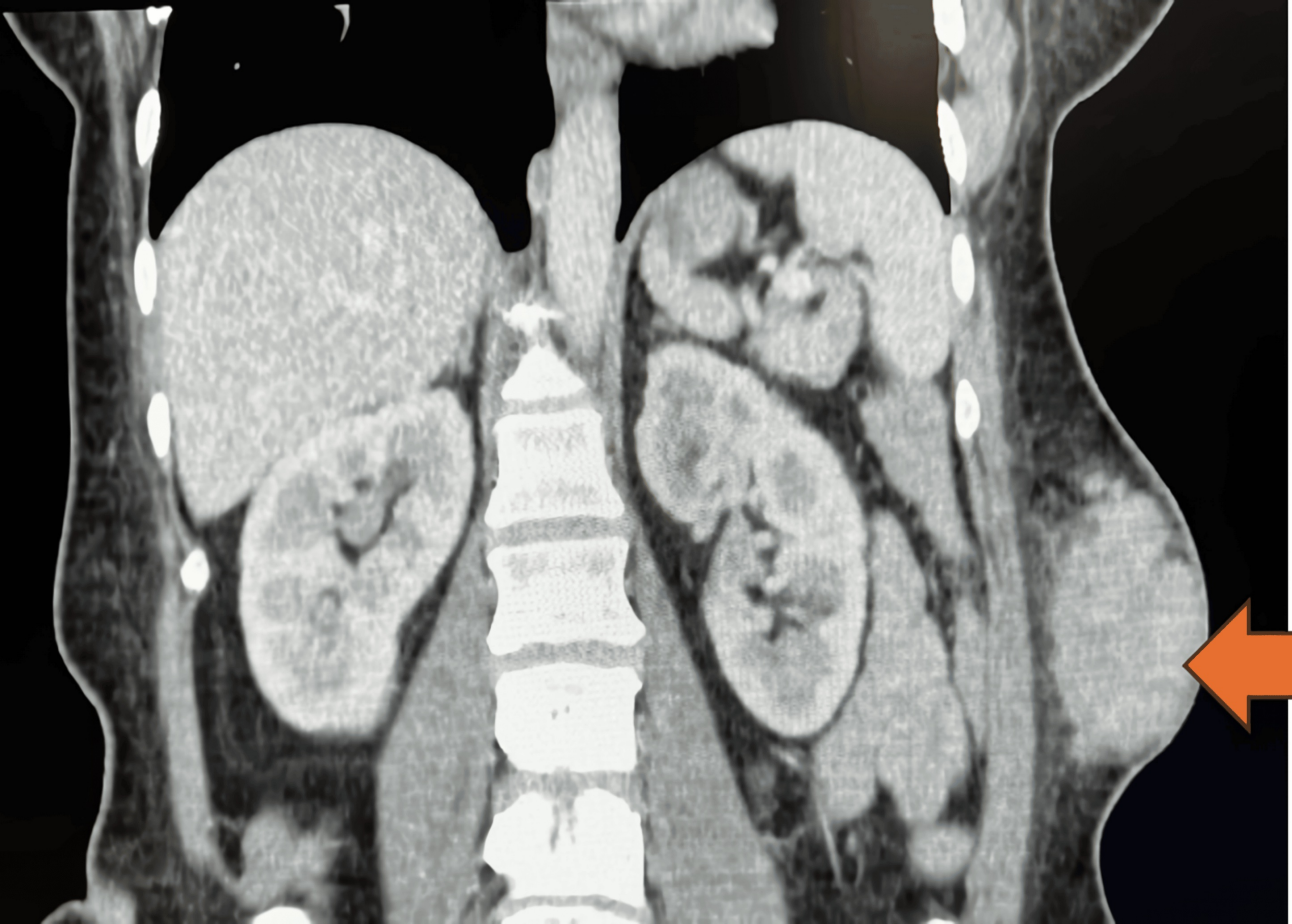
CT of abdomen (sagittal view): The orange arrow indicates the soft tissue mass on left flank

**Figure 2:**
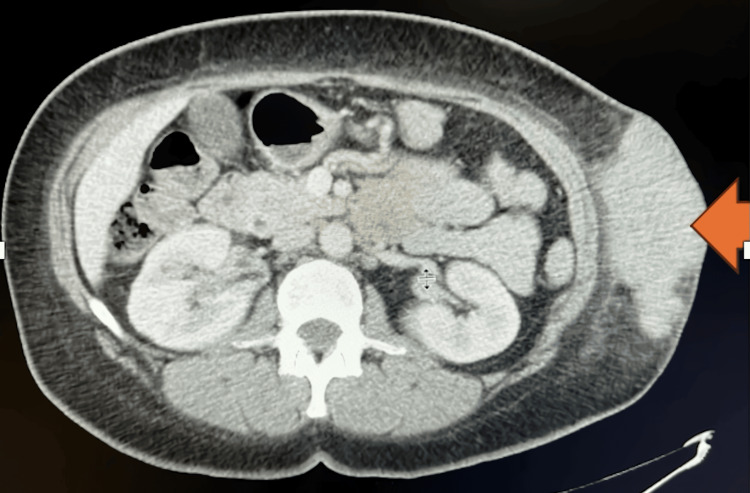
CT of abdomen (coronal view): the orange arrow indicates the soft tissue mass on left flank

A 50-year-old African-American female, with a past history of hypertension, diabetes mellitus, and hyperlipidemia, presented with a soft tissue mass located on the left flank (Figures 1, 2). The mass was located across the epigastric, umbilical, left hypochondrium, and left lumbar quadrants across the abdomen. The patient reported that the mass had been there for over 15 years. She stated that the mass did not grow and had remained asymptomatic all these years; however, the mass began to grow at a rapid rate in the last few months. She cannot recall any specific trigger that led to the rapid growth of the mass. Associated symptoms included weight loss, generalized fatigue, and loss of appetite. On physical exam, there were erythematous changes overlying the soft tissue mass, with no tenderness or pain. The mass had a smooth texture and felt firm to the touch. The patient's presenting symptoms of a skin mass were in contrast to classic RDD. She did not have any lymphadenopathy, which is seen in the majority of RDD cases.

**Figure 3:**
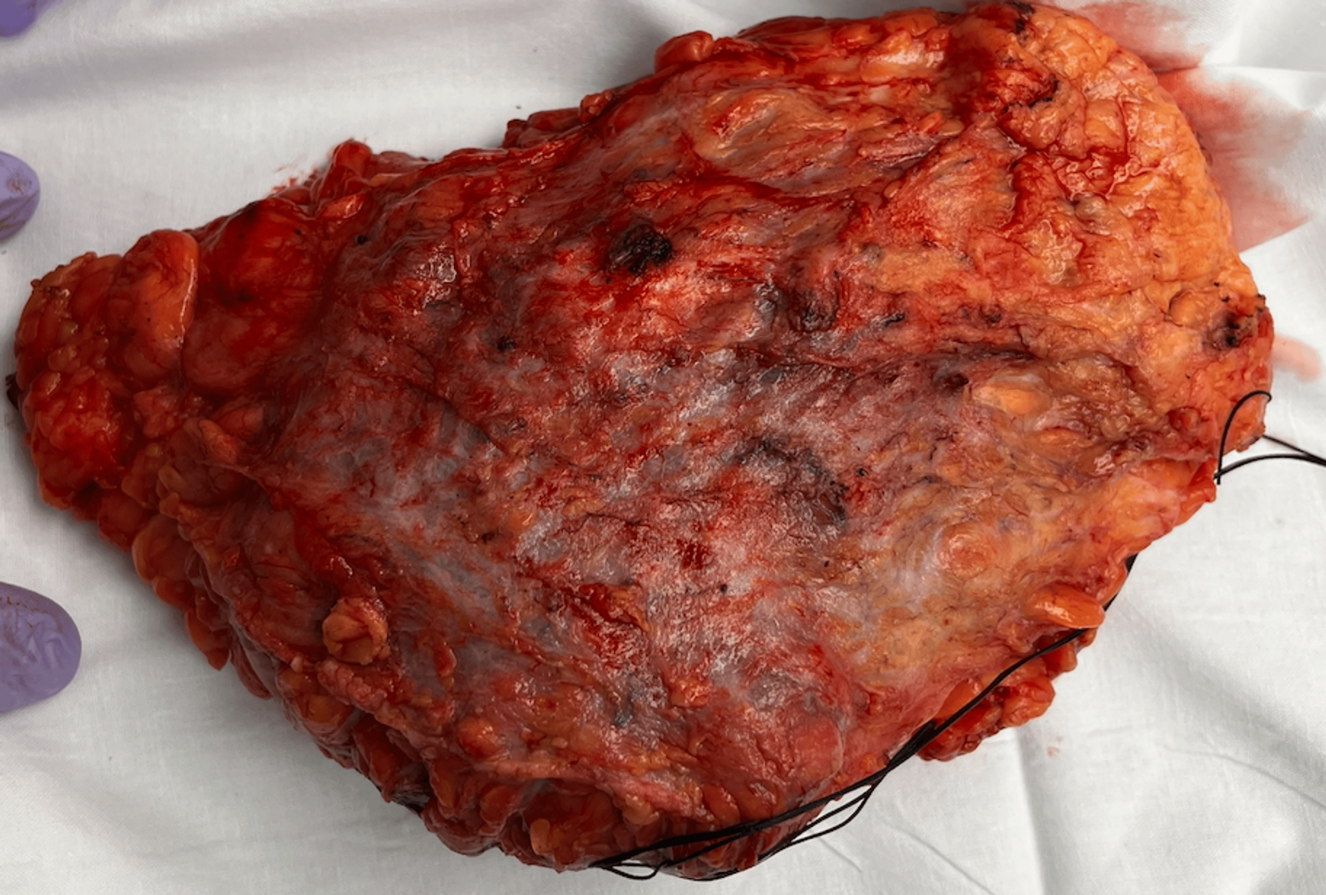
Pathology image of mass from surgical excision.

Based on the patient’s history and physical exam, a cancer diagnosis was excluded. She underwent a core needle biopsy. The pathology report for the specimen found a nodular, pink-white, rubbery, firm mass that measured 21 x 11.5 x 6 cm (Figure 3). According to the pathology report, histological analysis showed proliferation of epithelioid histiocytes and mixed chronic inflammation, consisting of neutrophils, lymphocytes, and macrophages. The immunohistochemistry findings were positive for CD68, CD163, Cyclin D1, and OCT-2. Immunohistochemistry stained strongly positive for EMA and ALK1 and weakly positive for DOG1 and S100 (Table 1); as a result, a diagnosis of cutaneous RDD was confirmed. S-100 is a key marker for histiocytes and is expressed in the majority of RDD cases. CD68 and CD163 highlight the histiocytic cells within the lesions [8]. After further discussion, the patient decided to undergo a wide, surgical local excision, with complete removal of the mass. The pathology report from the surgical excision supported previous results from the core needle biopsy. She achieved complete resolution of symptoms with no recurrence.

**Table 1:** Immunohistochemistry from pathology report showing results from staining. Rosai-Dorfman disease histiocytes are typically positive for EMA, ALK1, and S100

STAIN	INTERPRETATION
SMA	Negative
MSA	Negative
DESMIN	Negative
CK-PAN	Negative
EMA	1+, Cytoplasmic granular
ALK1	2+, Cytoplasmic
CD117	Negative
DOG1	Weak staining, favor nonspecific staining
S100	Zonal bluish positivity, favor nonspecific staining

## Discussion

A larger part of the discussion regarding further study of RDD will be mentioned in this section, which will focus on the various clinical manifestations, treatment options, role of immunohistochemistry, and potential opportunities for misdiagnosis.

RDD has a wide range of clinical variability. Classic RDD affects the cervical, mediastinal, axillary, and inguinal lymph nodes. Skin masses (extra-nodal RDD) are seen more commonly in children [9]. Thus, this case is unique because a middle-aged woman presented with a soft tissue mass with no associated lymphadenopathy.

Cancers, such as lymphomas, can present very similarly with enlarged, painless cervical lymphadenopathy. On the other hand, nasopharyngeal involvement can present with recurrent sinusitis [8]. Cutaneous involvement can present with masses mimicking lipomas [9]. Central mediastinum involvement can present with facial swelling and pleural effusions, mimicking interstitial lung diseases and tumors [10]. CNS involvement can manifest with symptoms of headache, motor/sensory abnormalities, and cranial nerve deficits [10]. Bone manifestations can present with bone lesions, mimicking sarcomas [10]. Thus, further research needs to be conducted to further classify the wide manifestations of RDD to create further clinical knowledge and awareness, so other mimicking, serious pathologies can be effectively ruled out.

Treatment options also need to be studied to avoid unnecessary invasive procedures. It is crucial to utilize immunohistochemistry markers. These markers provide definite diagnosis, which can provide reassurance to the patient and avoid aggressive procedures, such as chemotherapy or radiation. It has been reported that 80% of RDD cases were found to spontaneously self-resolve; once the trigger has been removed from the lymph node, the lymphadenopathy self-resolves [9]. Common triggers include any condition that induces an inflammatory response, such as an infection [9]. In other cases, treatments can include, but are not limited to, immunosuppressants, chemotherapy, radiation, surgery, and biologics. Due to the wide range of treatment options available and uncertainty of etiology of the disease, managing the treatment for RDD is very individualized for each patient. Patients may opt to follow symptoms with regular checkups with providers and await spontaneous resolution. Patients may also opt to pursue aggressive measures, such as surgical procedures or chemotherapy.

On the other hand, failure to adequately deploy measures to diagnose RDD can often lead to misdiagnosis. For example, this patient’s soft tissue mass could easily have been misdiagnosed as a lipoma, since it resembled one on physical exam. However, accurate measures, such as an excisional biopsy, should be done to assess pathology to effectively rule out malignancy and guide treatment.

Overall, further research needs to focus on categorizing clinical algorithms and patterns of the wide manifestations of RDD, which can then effectively lead to precise disease management for the welfare of the patients’ overall quality of health.

## Conclusions

The presented case report discusses the importance of diagnosing RDD as well as understanding the clinical manifestations of the disease to further advance clinical management. Given the diverse range of clinical manifestations RDD can exhibit, it is crucial to maintain a high level of clinical suspicion when patients present with generalized lymphadenopathy. Additionally, extra-nodal symptoms, such as cutaneous involvement, should also raise suspicion for RDD. Because RDD is so rare, it is often overlooked, partly due to the absence of clinical algorithms for classifying its subtypes and guiding management. Therefore, expanding knowledge in this area is crucial to improving the management of RDD and accurately differentiating it from conditions with similar presentations. Having clear guidelines for RDD management can help minimize invasive procedures by providing standardized diagnostic criteria and treatment protocols, ensuring that only necessary interventions are performed while avoiding unnecessary invasive tests.
